# Draft Genome Sequences of *Legionella* Presumptive Novel Species Isolated during Environmental Surveillance in Artificial Water Systems

**DOI:** 10.1128/MRA.00307-21

**Published:** 2021-05-13

**Authors:** Luna Girolamini, Silvano Salaris, Massimiliano Orsini, Maria Rosaria Pascale, Marta Mazzotta, Antonella Grottola, Sandra Cristino

**Affiliations:** a Department of Biological, Geological, and Environmental Sciences, University of Bologna, Bologna, Italy; b Laboratory of Microbial Ecology and Genomics of Microorganisms, Istituto Zooprofilattico Sperimentale delle Venezie, Legnaro, Italy; c Regional Reference Laboratory for Clinical Diagnosis of Legionellosis, Unit of Microbiology and Virology, Modena University Hospital, Modena, Italy; Loyola University Chicago

## Abstract

We present the draft genome sequences of three *Legionella* strains that were isolated from a hotel water distribution system. *Legionella* species identification was performed by macrophage infectivity potentiator (*mip*) and RNA polymerase β subunit (*rpoB*) gene sequencing. Whole-genome sequencing and average nucleotide identity results supported the hypothesis of new *Legionella* species isolation.

## ANNOUNCEMENT

The *Legionella* genus contains pathogenic Gram-negative bacteria that are ubiquitous in soil and water environments. It consists of more than 60 species, all of them potentially able to cause Legionnaires’ disease, a severe form of pneumonia (1).

The *Legionella* sp. strains 27fs60 (S60), 30fs61 (S61), and 30cs62 (S62) were isolated from three different samples from a hotel’s hot water distribution system in the Emilia-Romagna region (Italy) during a routine *Legionella* surveillance program. Water sampling and *Legionella* isolation were performed according to ISO 19458:2006 and ISO 11731:2017, respectively (2, 3). Samples were seeded onto selective medium with glycine-vancomycin-polymyxin B-cycloheximide (GVPC) and were incubated for 15 days at 35°C ± 2°C in 2.5% CO_2_. Suspected colonies were subcultured on buffered charcoal yeast extract (BCYE) without l-cysteine (Thermo Fisher Scientific, Basingstoke, UK).

The DNA was extracted with InstaGene matrix (Bio-Rad, Hercules, CA, USA), and identification of isolates was performed by macrophage infectivity potentiator (*mip*) and RNA polymerase β subunit (*rpoB*) gene sequencing (4, 5). Amplicons were sequenced using BigDye chemistry and analyzed on an ABI Prism 3100 genetic analyzer (Applied Biosystems, Foster City, CA, USA). The *mip* sequences were compared with the European Working Group for *Legionella* Infections (EWGLI) database. A BLAST search of the NCBI database was carried out for both *mip* and *rpoB* gene sequences. The best match returned was Legionella quateirensis reference strain ATCC 49507 (GenBank accession number GCA_001467955.1), with similarities of 98.45% and 94.8% for *mip* and *rpoB*, respectively.

One hundred nanograms of genomic DNA was used for next-generation sequencing (NGS) library preparation using the Illumina Nextera XT DNA library preparation kit (New England Biolabs, Ipswich, MA, USA). Sequencing was performed on the Illumina NextSeq 500 platform (2 × 150-bp paired-end reads). Raw reads were used as input data for TORMES v.1.2.0 (6), an automated pipeline for analysis of whole bacterial genomes. TORMES includes sequence quality filtering (PRINSEQ v.0.20.4) (7) and *de novo* genome assembly (SPAdes v.13.4.1) (8), as well as other downstream analyses not used for our purpose. Scaffolding was performed using TORMES contigs as input for CSAR v.1.1.1 (9) with an evolutionarily related reference genome, i.e., Legionella fallonii (GenBank accession number NZ_LN614827.1) The final assemblies were further improved using Geneious Prime v.2020.2.4 software (10) and were submitted to GenBank with annotation by the NCBI Prokaryotic Genome Annotation Pipeline (PGAP) v.4.3 (11). Default parameters were used for all software tools unless otherwise noted. Table 1 summarizes results from assembly and annotation by the PGAP and the completeness of genome assembly determined by Benchmarking Universal Single-Copy Orthologs (BUSCO) v.5.0.0 (12).

**TABLE 1:** Genome statistics from NCBI and BUSCO quality analyses

Attribute	Data for strain:		
	27fs60 (S60)	30fs61 (S61)	30cs62 (S62)
No. of raw reads	4,184,062	3,851,726	3,626,424
Avg read length (bp)	149	149	149
Coverage (×)	142	131	124
Total length (bp)	4,211,919	3,709,497	4,136,543
No. of contigs	23	37	32
GC content (%)	39.00	39.10	39.00
*N*_50_ (bp)	312,097	166,809	176,017
No. of coding sequences	3,542	3,155	3,491
No. of rRNAs	3	3	3
No. of tRNAs	41	37	39
BUSCO results (% [no. of genes])
Complete	99.2 (123)	93.5 (116)	95.2 (118)
Single-copy complete	99.2 (123)	93.5 (116)	95.2 (118)
Duplicated complete	0.0 (0)	0.0 (0)	0.0 (0)
Fragmented	0.8 (1)	0.8 (1)	0.8 (1)
Missing	0 (0)	5.7 (7)	4.0 (5)
Total no. of BUSCO genes	124	124	124

The FastANI tool (13) was used to compare the average nucleotide identity (ANI) of the three strains against 1,009 *Legionella* sequences that had been downloaded from the NCBI database using the ncbi-genome-download tool (https://github.com/kblin/ncbi-genome-download). FastANI identified the closest relative of strain S60 to be L. quateirensis NCTC 12376 (GenBank accession number GCA_900452695.1) (91.31%) and the closest relative of strains S61 and S62 to be L. quateirensis ATCC 49507 (91.45% and 91.44%, respectively). Since the assumption is that two strains showing pairwise ANI values below a given threshold (95% or 96 %) belong to different species (14), our results led us to consider these strains new species.

Studying the whole genome allows investigators to better identify already known species and to discover new ones, improving the knowledge of the ecological, virulence, and resistance characteristics of *Legionella*.

### Data availability.

The draft genome assemblies are available in the GenBank database and can be accessed with SRA and assembly accession numbers SRP292355 and JADOBG000000000 (S60), SRP295125 and JADWVM000000000 (S61), and SRP295130 and JADWVN000000000 (S62).
